# The transcription factor scleraxis is a critical regulator of cardiac fibroblast phenotype

**DOI:** 10.1186/s12915-016-0243-8

**Published:** 2016-03-17

**Authors:** Rushita A. Bagchi, Patricia Roche, Nina Aroutiounova, Leon Espira, Bernard Abrenica, Ronen Schweitzer, Michael P. Czubryt

**Affiliations:** Institute of Cardiovascular Sciences, Boniface Hospital Albrechtsen Research Centre and Department of Physiology and Pathophysiology, University of Manitoba, R4008 St. Boniface Hospital Albrechtsen Research Centre, 351 Tache Avenue, Winnipeg, MB R2H 2A6 Canada; Shriners Hospital for Children, Research Division and Department of Cell and Developmental Biology, Oregon Health and Science University, Portland, OR 97239 USA

**Keywords:** Fibroblast, Myofibroblast, Gene expression, Phenoconversion, Transcription, Extracellular matrix, EMT

## Abstract

**Background:**

Resident fibroblasts synthesize the cardiac extracellular matrix, and can undergo phenotype conversion to myofibroblasts to augment matrix production, impairing function and contributing to organ failure. A significant gap in our understanding of the transcriptional regulation of these processes exists. Given the key role of this phenotype conversion in fibrotic disease, the identification of such novel transcriptional regulators may yield new targets for therapies for fibrosis.

**Results:**

Using explanted primary cardiac fibroblasts in gain- and loss-of-function studies, we found that scleraxis critically controls cardiac fibroblast/myofibroblast phenotype by direct transcriptional regulation of myriad genes that effectively define these cells, including extracellular matrix components and α-smooth muscle actin. Scleraxis furthermore potentiated the TGFβ/Smad3 signaling pathway, a key regulator of myofibroblast conversion, by facilitating transcription complex formation. While scleraxis promoted fibroblast to myofibroblast conversion, loss of scleraxis attenuated myofibroblast function and gene expression. These results were confirmed in scleraxis knockout mice, which were cardiac matrix-deficient and lost ~50 % of their complement of cardiac fibroblasts, with evidence of impaired epithelial-to-mesenchymal transition (EMT). Scleraxis directly transactivated several EMT marker genes, and was sufficient to induce mesenchymal/fibroblast phenotype conversion of A549 epithelial cells. Conversely, loss of scleraxis attenuated TGFβ-induced EMT marker expression.

**Conclusions:**

Our results demonstrate that scleraxis is a novel and potent regulator of cellular progression along the continuum culminating in the cardiac myofibroblast phenotype. Scleraxis was both sufficient to drive conversion, and required for full conversion to occur. Scleraxis fulfills this role by direct transcriptional regulation of key target genes, and by facilitating TGFβ/Smad signaling. Given the key role of fibroblast to myofibroblast conversion in fibrotic diseases in the heart and other tissue types, scleraxis may be an important target for therapeutic development.

**Electronic supplementary material:**

The online version of this article (doi:10.1186/s12915-016-0243-8) contains supplementary material, which is available to authorized users.

## Background

Resident cardiac fibroblasts (FBs) are active regulators of myocardial function in health and disease, serving as a source of autocrine and paracrine factors to support cardiomyocyte function, as well as synthesizing the extracellular matrix (ECM)—a highly dynamic structure necessary for maintaining cardiac homeostasis, transmitting contractile forces, preventing myocyte slippage and facilitating intercellular signaling [1]. Cardiomyocytes comprise the bulk of the myocardial volume; however, interstitial FBs are the most numerous cell type and coordinate the maintenance of ECM through production and secretion of structural proteins, growth factors, cytokines and proteinases [2].

While FB activity is typically low in the healthy heart, aging, stress, inflammation or damage can instigate a phenotypic conversion to proto-myofibroblasts (pMFBs) and myofibroblasts (MFBs), resulting in dramatic up-regulation of ECM synthesis, particularly fibrillar collagen types I and III [1, 3–6]. During this transition, FBs begin to express α-smooth muscle actin (αSMA/Acta2), which is incorporated into newly formed contractile bundles and stress fibers that impart cell contractility to facilitate damage repair [7–9]. MFBs are further distinguished from their precursors by up-regulation of non-muscle myosin heavy chain IIB (embryonic smooth muscle myosin; SM_emb_/Myh10) and the extra domain-A splice variant of fibronectin (EDA-Fn/Fn1) [3, 10, 11]. Production and secretion of factors involved in cell signaling and adhesion, including proteoglycans and matricellular proteins, also increase [12–14]. In the non-stressed heart, MFBs are found at the cardiac valves, playing a key role in valve maintenance [15]. Several inducers of the FB to MFB conversion process have been identified, including TGFβ; however, the underlying molecular mechanisms remain incompletely defined, and it is unclear whether FB phenotype conversion is controlled by a central factor [10, 16].

The basic helix-loop-helix transcription factor scleraxis (Scx) is required for development and function of ECM-rich tissues, including tendons and cardiac valves [15, 17]. We have shown that Scx directly regulates *Col1a2* gene expression in primary cardiac FBs and MFBs, and is up-regulated in the cardiac infarct scar or in response to TGFβ signaling [6, 18]. Scx similarly controls expression of *Col1a1* in tenocytes, suggesting a direct role for this transcription factor in modulating fibrillar collagen production across tissues [19]. However, it is unknown whether Scx plays a broader role in ECM synthesis or FB biology.

Here we report that Scx is a required and potent regulator of the cardiac FB and MFB phenotype and attendant gene expression, including the hallmarks of ECM production and cell contraction. *Ex vivo*, Scx induced the expression of various fibrillar collagens, proteoglycans, matrix metalloproteinases (MMPs) and numerous markers of the FB and MFB phenotype in primary cardiac pMFBs, and directly transactivated the gene promoters of vimentin, MMP2 and fibronectin. Scx was sufficient and necessary for the expression of αSMA via direct promoter binding, inducing the incorporation of αSMA into stress fibers and consequent cell contraction. In contrast, Scx knockdown significantly attenuated the expression of ECM and FB/MFB marker genes, and fully attenuated TGFβ-mediated cell contraction. These findings were recapitulated *in vivo*: the hearts of Scx knockout mice presented with dramatic losses of ECM mass and expression of ECM and FB marker genes. The function of Scx null cardiac FBs could be rescued by restoration of Scx expression. We found that Scx was required for Smad3-mediated fibrillar collagen gene expression, and the assembly of a Smad3-containing transcriptional complex at the *Col1a2* gene promoter. Surprisingly, Scx null hearts exhibited a ~50 % reduction in FB number. This effect may be due to a failure of epithelial precursors to undergo mesenchymal transition during development since Scx was found to regulate expression of mesenchymal markers, including *Twist1* and *Snai1*, leading to elevated epithelial and reduced mesenchymal marker gene expression in Scx null hearts. Furthermore, Scx expression in A549 epithelial cells reduced epithelial markers while up-regulating mesenchymal and FB markers, as well as fibrillar collagens. Together, these results reveal a novel and critical role for Scx in governing the hallmarks of the cardiac FB phenotype, including the regulation of mesenchymal character, conversion to MFBs, ECM synthesis and cell contraction.

## Results

### Scleraxis is sufficient and necessary for induction of cardiac ECM gene expression

**Fig. 1:**
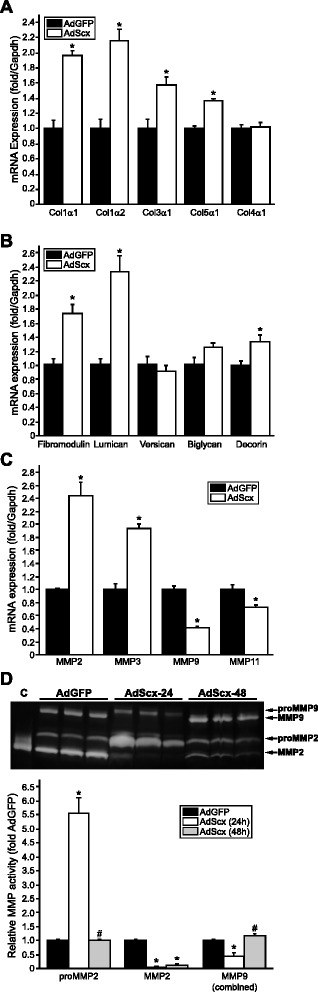
Scleraxis up-regulates matrix target genes. **a**–**c** Assay of collagen (**a**), proteoglycan (**b**) and matrix metalloproteinase (**c**) mRNA expression by qPCR following over-expression of Scx (AdScx) in primary rat cardiac proto-myofibroblasts compared to controls (AdGFP) reveals numerous matrix genes are induced; *n* = 3. **d** Protein lysates from AdGFP- or AdScx-infected primary rat cardiac proto-myofibroblasts were assayed for MMP activity by gel zymography. Zymograms were obtained 24 or 48 h following infection and revealed transient induction of proMMP2 and loss of MMP9 following Scx over-expression; C, control recombinant human MMP2 protein; *n* = 3. *P < 0.05 vs AdGFP, #*P* < 0.05 vs corresponding 24 h time point

We previously demonstrated that Scx directly regulates the expression of *Col1a2*, one of two component chains of type I collagen, in cardiac MFBs *in vitro* via conserved promoter E-boxes, and others have shown that Scx up-regulates the *Col1a1* chain in tenocytes [19]. However, while type I collagen is the primary component of the cardiac ECM, it is unclear whether Scx plays a broader regulatory role in ECM gene expression, thus we examined this possibility using gain- and loss-of-function approaches. In isolated primary rat pMFBs, an intermediate phenotype between FBs and MFBs [3, 12, 16], Scx induced expression of the major cardiac fibrillar collagens (*Col1a1*, *Col1a2*, *Col3a1*, *Col5a1*), but not non-fibrillar *Col4a1* (Fig. 1a), suggesting that fibrillar collagens were specifically impacted, and that our results do not represent a general effect on collagen expression. Several proteoglycans were up-regulated by Scx, including *Fmod*, *Lum* and *Dcn* (Fig. 1b), similar to results reported for proteoglycan regulation by Scx in cardiac valves [20].

Scx over-expression significantly increased *Mmp2* and *Mmp3* expression, but decreased *Mmp9* and *Mmp11* (Fig. 1c). In agreement with these results, Scx transiently increased proMMP2 activity by over fivefold (concomitant with a loss of mature MMP2 activity), but decreased combined MMP9/proMMP9 activity within 24 h (Fig. 1d). By 48 h, proMMP2 and combined MMP9 activity returned to control levels. Some MMPs (e.g. *Mmp2*, *Mmp3*) may thus be under direct transcriptional control by Scx.

**Fig. 2:**
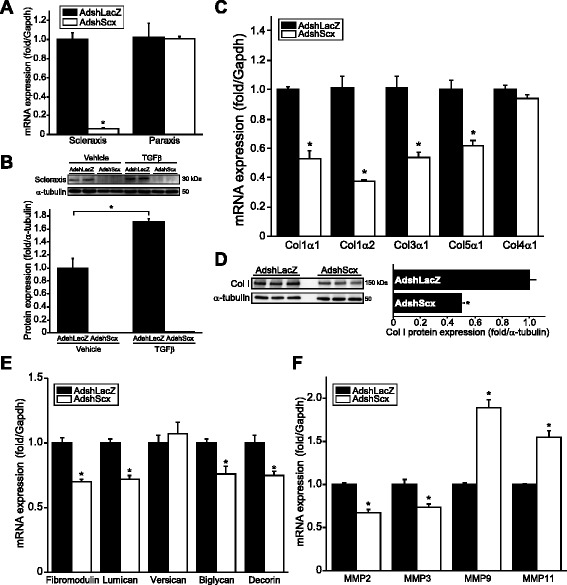
Cardiac matrix gene expression is attenuated by scleraxis knockdown. **a** Primary cardiac proto-myofibroblasts exhibited loss of Scx but not paraxis mRNA following infection with adenovirus encoding an shRNA targeting Scx (AdshScx) but not control shRNA (AdshLacZ) for 72 h (assayed by qPCR). Results were normalized to the respective AdshLacZ sample; *n* = 3. **b** Cells treated as in (**a**), with or without 10 ng/mL TGFβ or vehicle, were assayed for Scx protein expression 72 h after infection and results normalized to α-tubulin; *n* = 4. AdshScx attenuated Scx protein expression even in the presence of TGFβ. **c**–**d** Fibrillar collagen mRNA (**c**) and nascent soluble 150 kDa collagen I protein (**d**) expression was down-regulated following Scx knockdown (assayed by qPCR or western blotting, respectively), with cell treatment and normalization as in (**a**); *n* = 3. **e**–**f** The expression of mRNAs encoding several proteoglycans (**e**) and matrix metalloproteinases (**f**) (assessed by qPCR) was reduced following Scx knockdown; *n* = 3. *P < 0.05 vs corresponding AdshLacZ control

We confirmed that Scx is required for ECM gene expression via shRNA-mediated loss-of-function in primary rat cardiac pMFBs. We generated a novel adenovirus encoding a Scx shRNA (AdshScx), which rapidly and potently attenuated Scx expression but had no effect on paraxis, a transcription factor with the highest homology to Scx (Fig. 2a) [21]. Scx expression was lost even after treatment with TGFβ, which we have shown potently induces Scx expression (Fig. 2b) [6, 18]. Scx knockdown attenuated expression of the same fibrillar collagens induced by Scx over-expression, and did not affect *Col4a1* expression (Fig. 2c, d). Scx knockdown similarly reduced expression of several proteoglycans, including *Fmod*, *Lum* and *Dcn*, complementing the over-expression results (Fig. 2e). Scx loss reduced *Mmp2* and *Mmp3* expression, while inducing *Mmp9* and *Mmp11* (Fig. 2f). Together, these results indicate that Scx exerts broad control of genes regulating ECM synthesis and turn-over.

### Cardiac fibroblast phenotype regulation by scleraxis

**Fig. 3:**
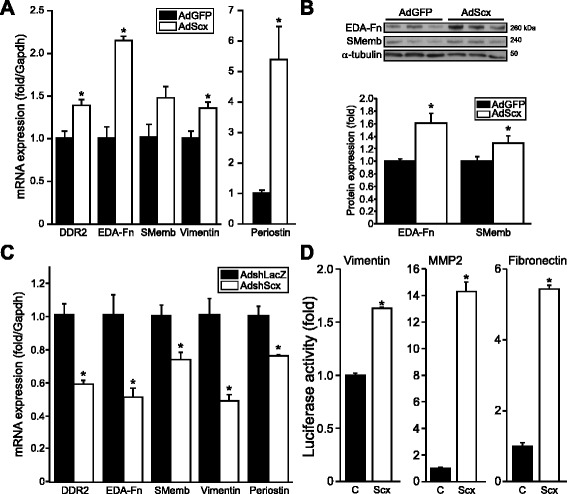
Scleraxis regulates cardiac fibroblast phenotype. **a**–**c** Fibroblast/myofibroblast marker gene mRNA (**a**, **c**) and protein (**b**) expression was assayed in primary cardiac proto-myofibroblasts following Scx over-expression (AdScx) vs controls (AdGFP) (**a**, **b**), or following Scx knockdown (AdshScx) compared to controls (AdshLacZ) (**c**), assayed by qPCR or western blot; *n* = 3. Scx loss induced similar down-regulation of fibroblast and myofibroblast markers, while Scx over-expression induced marker expression. **d** Scx transactived the vimentin, MMP2 and fibronectin gene promoters compared to empty vector control (C) as determined by luciferase reporter assays in NIH-3T3 fibroblasts; *n* = 3 (vimentin, fibronectin) or *n* = 4 (MMP2). *P < 0.05 vs AdGFP (**a**, **b**), vs AdshLacZ (**c**) or vs control empty vector (**d**)

Increased ECM expression is a hallmark of FB to MFB phenotype conversion. Given Scx’s regulation of a broad variety of ECM genes, we hypothesized that Scx governs cardiac FB phenotype, thus we assayed a broad panel of FB and MFB markers following Scx gain- or loss-of-function. Scx over-expression in cardiac pMFBs increased synthesis of nearly all markers tested (Fig. 3a, b), including the collagen receptor *DDR2*, EDA-Fn/*Fn1*, vimentin/*Vim* and periostin/*Postn*, an integrin ligand implicated in fibrosis of various tissues and cardiac FB specification [22, 23]. Scx knockdown conversely decreased all markers (Fig. 3c), suggesting reversion away from the MFB phenotype. Luciferase assay demonstrated that Scx significantly transactivated the *Vim*, *Mmp2* and *Fn1* gene promoters (Fig. 3d), supporting that Scx’s effects are primarily due to direct target gene transactivation rather than by a secondary mediator.

Scx regulates target gene expression via interaction with DNA promoter *cis* E-box elements [6, 18]. We previously showed that Scx’s basic DNA-binding and helix-loop-helix protein-binding domains are functionally critical, with deletion of the basic domain resulting in a dominant negative mutant [6, 18]. A Scx mutant lacking both domains (ScxΔΔ) had no effect on transactivation of the human *COL1A2* promoter, failed to attenuate Scx-mediated transactivation and did not alter the expression of fibrillar collagens or FB/MFB markers in pMFBs, suggesting that this double mutant is inactive, and indicating that the transcriptional activity of Scx is required for regulating target gene expression (Additional file 1: Figure S1).

### Scleraxis directly regulates αSMA gene expression

**Fig. 4:**
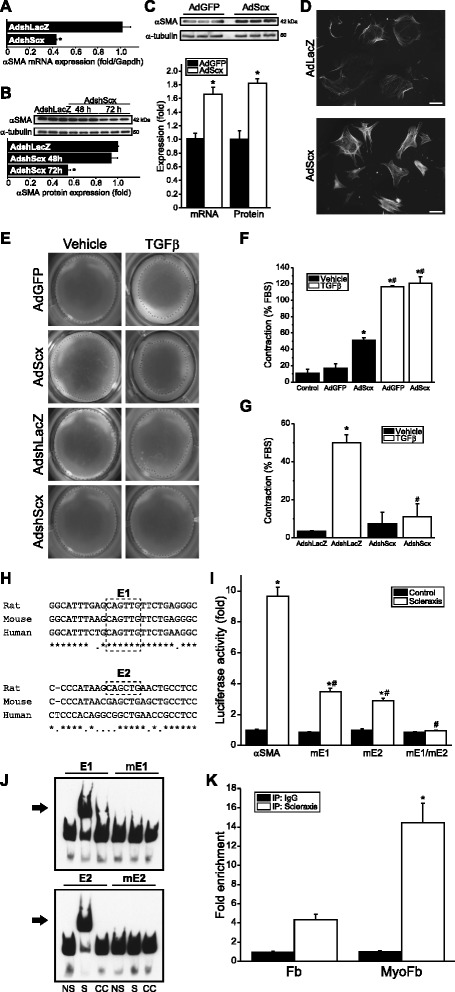
Scleraxis regulates cardiac myofibroblast α-smooth muscle actin expression and cell contraction. **a**–**b** αSMA mRNA (**a**) and protein (**b**) expression were assayed by qPCR or western blot, respectively, following knockdown of Scx (AdshScx) in primary cardiac proto-myofibroblasts, showing attenuated αSMA expression compared to control (AdshLacZ); *n* = 3. **c** Scx over-expression (AdScx) up-regulated αSMA expression in primary rat cardiac proto-myofibroblasts as determined by mRNA or protein expression (qPCR or western blot, respectively); *n* = 3. **d** Induced αSMA was incorporated into stress fibers, as demonstrated by immunocytochemistry, indicating promotion of the myofibroblast phenotype by Scx; 20× objective, scale bar = 65 μm. **e** Primary cardiac proto-myofibroblasts seeded onto compressible collagen gel matrices were assayed for gel contraction following Scx over-expression or knockdown, with or without concomitant 10 ng/mL TGFβ_1_ to induce contraction. Crosses denote the detected margin of the collagen gel. **f**–**g** Quantification of gel contraction images, reported as the percentage of the maximum contraction induced by 10 % FBS, demonstrates that Scx over-expression induces cell contraction (**f**), while Scx knockdown attenuates TGFβ_1_-induced contraction (**g**); n = 6 ((**f**) except AdGFP + TGFβ and AdScx + TGFβ; *n* = 3 each) or *n* = 3 (**g**). *P < 0.05 vs AdshLacZ (**a**–**b**), vs AdGFP (**c**, **f**) or vs AdshLacZ + vehicle (**g**), #*P* <0.05 vs vehicle controls (**f**) or vs AdshLacZ + TGFβ (**g**). **h** Alignment of putative Scx-binding E-boxes (E1 and E2; *dashed lines*) in the proximal rat, mouse and human αSMA promoters. Asterisks and periods denote nucleotides conserved across three or two species, respectively. **i** Mutation of one or both E-boxes (mE1, mE2) attenuates transactivation of the rat αSMA proximal promoter by Scx as determined by luciferase reporter assay; *n* = 3. **j** Electrophoretic mobility shift assays demonstrate Scx-binding to E-boxes E1 and E2; this binding is abolished when the E-boxes are mutated (mE1 and mE2). NS, non-shifted lane; S, shifted lane; CC, 500× cold competition. The arrow denotes the shifted complex. **k** Scx-binding to the αSMA gene promoter is significantly enriched in cardiac myofibroblasts (MyoFb) compared to fibroblasts (Fb) as determined by chromatin immunoprecipitation assay with qPCR quantification of Scx-bound DNA; *n* = 3. *P < 0.05 vs control empty vector (**i**) or vs Fb (**k**), #*P* < 0.05 vs scleraxis + non-mutated promoter (**i**)

Since Scx induced the MFB phenotype, we examined the effect of Scx on expression of αSMA/*Acta2*, a contractile protein not typically expressed in cardiac FBs, but which is sharply up-regulated during conversion to MFBs [3, 6]. Scx knockdown reduced (Fig. 4a, b) while over-expression induced αSMA expression (Fig. 4c) and incorporation into stress fibers (Fig. 4d), a characteristic of MFBs [12]. In agreement with this finding, Scx induced cell contraction of cardiac pMFBs (Fig. 4e, f). Intriguingly, knockdown of Scx completely attenuated cell contraction induced by TGFβ (Fig. 4e, g), demonstrating a requirement for Scx in this process.

We tested whether αSMA is a direct Scx gene target. The rat αSMA proximal promoter contains two well-characterized E-boxes critical for *cis* regulation (Fig. 4h) [24, 25]. Scx transactivated the αSMA promoter nearly tenfold, and mutating these E-boxes attenuated promoter transactivation by Scx (Fig. 4i). Electrophoretic mobility shift assay demonstrated that Scx forms complexes with the intact but not mutated E-boxes (Fig. 4j). Chromatin immunoprecipitation confirmed this interaction; however, due to their close proximity (32 nucleotide separation) we could not resolve whether Scx bound to one or both E-boxes. The amount of DNA-bound Scx increased by over threefold in cardiac MFBs compared to FBs (Fig. 4k), congruent with increased Scx-mediated αSMA expression during phenotype conversion (Fig. 4c, d). This data contrasts with a previous report in mesangial cells reporting that Scx was capable of binding only to the second E-box, resulting in an inhibitory effect on αSMA expression [26]. Our results demonstrate that Scx activity across cell types is highly variable and requires empirical testing, and suggests unique roles for Scx in specific tissues.

### Scleraxis is required for cardiac fibroblast gene expression *in vivo*

Our *ex vivo* data revealed that Scx regulates FB phenotype and ECM synthesis, thus we assessed its *in vivo* role using knockout mice. Scx loss induces cardiac morphological anomalies, including altered valve structure, rounding of the ventricles and involution of the apex, but the effect on myocardial structure and composition has not been reported. It is unclear whether such alterations are pathological; however, as noted previously [15], homozygous null pups were under-represented at birth. Of 438 pups born, we identified 42 (9.5 %) nulls (KO), 261 (59.0 %) heterozygotes and 139 (31.4 %) wild type (WT); furthermore, 10 KO (23.8 % of KO neonates) died prior to 6 weeks of age. KO pups were runted (body weight WT 20.2 ± 0.8 g, KO 12.2 ± 0.6; *n* = 9–12; *P* < 0.0001). We noted a significant ~30 % hypotrophy of the heart (heart weight:tibia length WT 5.68 ± 0.15 mg/mm, KO 4.16 ± 0.11; *n* = 17–20; *P* <0.0001). Despite this decreased relative size, echocardiography revealed largely normal function (Additional file 2: Table S1), in agreement with earlier data [15]. Left ventricular end diastolic diameter, normalized to tibia length, was modestly (~10 %) but significantly elevated in KO mice and endocardial velocity trended lower, suggesting the existence of early systolic dysfunction. Such modest changes seem unlikely to contribute to the elevated mortality in KO mice, thus the causes of pre- and post-natal mortality remain unclear.

**Fig. 5:**
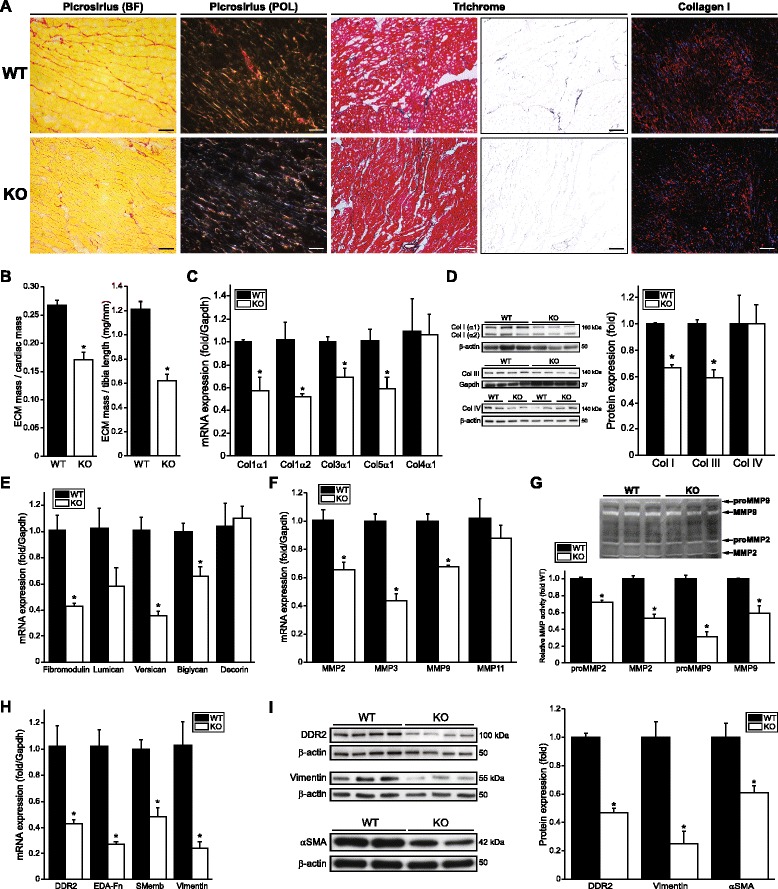
Scleraxis null mice exhibit reduced cardiac extracellular matrix and fibroblast marker gene expression. **a** Cross sections of hearts from wild type (WT) or Scx null (KO) hearts stained for fibrillar collagen using picrosirius red (BF, bright-field; POL, polarized light), Masson’s trichrome or via immunolabeling for total collagen I (*red*; plus DAPI nuclear staining in *blue*) revealed matrix loss in KO hearts; collagen I: 20× objective, scale bar = 35 μm; picrosirius/trichrome: 40× objective, scale bar = 66 μm. The blue color channel was extracted from the Masson’s trichrome sections for improved visualization. Samples are representative of at least three individual animals of each genotype. **b** Decellularized and dehydrated cardiac ventricular ECM from WT or KO mice was normalized to cardiac ventricular wet mass or tibia length and revealed ECM loss in KO hearts; *n* = 7 animals of each genotype. **c**–**d** Fibrillar collagen gene expression by qPCR (**c**) or western blotting normalized to *Gapdh* or β-actin (**d**) was decreased in Scx KO hearts compared to WT; *n* = 3 ((**c**) or collagen I blot in (**d**)) or *n* = 4 (collagen III and IV blots in (**d**)). Collagen I protein expression was the sum of the α1 and α2 isoforms. **e**–**f** The expression of mRNAs encoding several proteoglycans (**e**) and matrix metalloproteinases (**f**) (assessed by qPCR) was lost in Scx KO hearts; n = 3 animals of each genotype. **g** MMP2 and MMP9 activity (latent pro-form and mature, assayed by gel zymography) of protein lysates from WT or KO hearts was reduced in null animal hearts; *n* = 3 animals of each genotype. *P < 0.05 vs WT. **h**–**i** Fibroblast/myofibroblast marker gene mRNA (**h**) and protein (**i**) expression was assayed in WT and Scx KO mice, assayed by qPCR or western blot; *n* = 3 ((**h**) and vimentin in (**i**)) or *n* = 4 (DDR2 and αSMA in (**i**)). Scx loss induced similar down-regulation of fibroblast and myofibroblast markers. *P < 0.05 vs WT

We examined net fibrillar collagen expression in cardiac sections from WT or Scx KO mice histologically and by immunolabeling, and noted a clear reduction in collagen staining (Fig. 5a). Notably, collagen fibrils appeared diminished in number and size, and there was considerable reduction of perivascular collagen staining. The reduction in red/yellow fibers visible with picrosirius red staining under circularly polarized light is indicative of a net loss of organized collagen bundles (Fig. 5a). Cardiac ECM dry weight was reduced up to 49 % (Fig. 5b). Congruent with these data, mRNA and protein (Fig. 5c–d) levels of the major cardiac fibrillar collagens were dramatically decreased by 30–45 %. As in our *ex vivo* data, non-fibrillar *Col4a1* expression was unaltered.

Also in agreement with our *ex vivo* data, ECM alterations were not limited to collagens: we observed a general decrease in proteoglycan expression in the hearts of Scx KO mice compared to WT, with significant loss of *Fmod*, *Vcan* and *Bgn* (Fig. 5e). Conversely, decorin expression was undisturbed, thus proteoglycan loss was also not universal.

Similar to our *ex vivo* data, KO hearts exhibited significantly reduced mRNA expression of *Mmp2*, *Mmp3* and *Mmp9* (Fig. 5f). This down-regulation correlated with a generalized loss of activity of both the mature and latent (pro) forms of MMP2 and MMP9 (Fig. 5g). Scx gene deletion *in vivo* thus resulted in altered expression of collagen, proteoglycans and MMPs, which closely recapitulated our knockdown data.

Scx regulated cardiac FB phenotype *ex vivo* (Figs. 3, 4), thus we assayed relevant marker expression in WT and KO hearts. All markers tested were significantly down-regulated by over 50 % in Scx KO mice (Fig. 5h, i). In agreement with our data demonstrating direct regulation of αSMA expression by Scx (Fig. 4), we also observed a significant loss of αSMA protein in Scx KO hearts (Fig. 5i). Since αSMA is not expressed in cardiac FBs in the healthy heart, this loss likely reflects changes in vascular smooth muscle cells.

### Scleraxis is required for Smad3-mediated gene expression

**Fig. 6:**
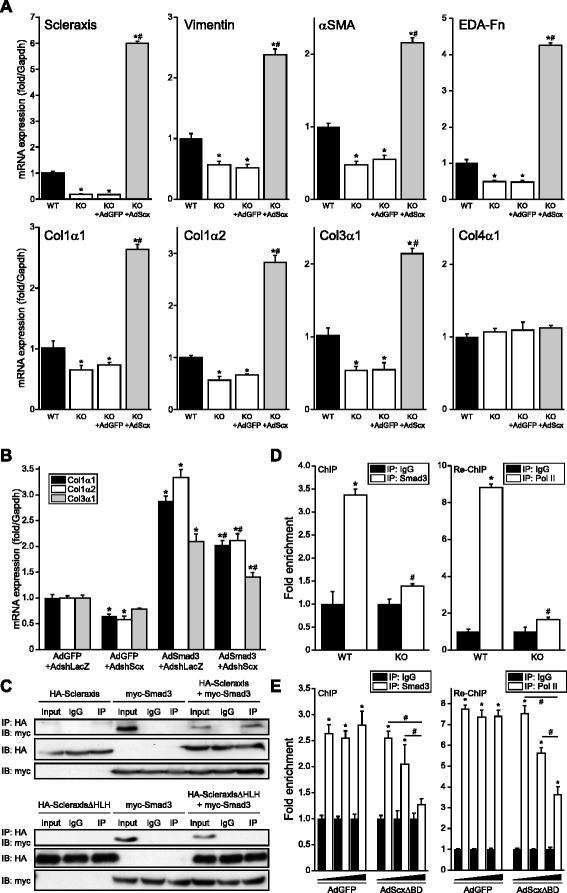
Requirement of scleraxis for Smad3-mediated gene expression. **a** Over-expression of Scx (AdScx) for 24 h rescues expression of vimentin, αSMA, ED-A fibronectin and fibrillar collagen mRNA (by qPCR) in primary cardiac proto-myofibroblasts obtained from Scx null mice compared to controls (AdGFP); *n* = 3 independent samples per genotype. Impaired collagen expression in Scx null cells is thus due to Scx loss and not a defect in the basal transcriptional machinery. **b** Up-regulation of fibrillar collagen mRNA expression by Smad3 (assessed by qPCR) is attenuated following Scx knockdown (AdshScx) compared to shRNA control in cardiac proto-myofibroblasts (AdshLacZ); *n* = 3. **c** Scx and Smad3 physically interact. Expression vectors for HA-tagged Scx and myc-tagged Smad3 were individually or jointly transfected into NIH-3T3 fibroblasts, then immunoprecipitated (IP) by anti-HA antibodies and subjected to western blotting with anti-myc antibodies (*upper panels*). Non-immunoprecipitated input and non-specific IgG antibodies were employed as positive and negative controls, respectively. Equal loading of transfected whole-cell lysates is shown by anti-HA and anti-myc western blots. Conversely, co-immunoprecipitation with Smad3 was not observed when a Scx mutant lacking its protein-interaction domain (HA-ScleraxisΔHLH) was employed (*lower panels*). **d** Smad3 and RNA polymerase II interaction with the *Col1α2* gene promoter is significantly impaired in cardiac fibroblasts derived from Scx KO mouse hearts compared to WT, as assessed by chromatin immunoprecipitation (anti-Smad3 antibody vs non-specific IgG antibody) and re-ChIP (anti-RNA Pol II antibody vs non-specific IgG antibody) followed by qPCR, indicating that Scx loss impairs Smad3 signaling and Smad3-mediated recruitment of the transcription complex to the *Col1α2* gene promoter; *n* = 3 independent samples per genotype. **e** Adenovirus-mediated over-expression of a DNA-binding Scx mutant (AdScxΔBD; 10, 50 or 100 MOI) in human ventricular myofibroblasts attenuates Smad3 and RNA polymerase II recruitment to the Smad-binding element of the *Col1α2* gene promoter compared to control (AdGFP) performed as in (**d**); *n* = 3. *P < 0.05 vs WT (**a**) or vs AdGFP + AdshLacZ (**b**) or vs IgG (**d**–**e**), #*P* < 0.05 vs KO + AdGFP (**a**) or vs AdSmad3 + AdshLacZ (**b**) or vs WT (**d**) or as indicated (**e**)

It is unclear whether the *in vivo* alterations in cardiac FB gene expression reflect an inherent transcription defect in these cells, thus we tested whether Scx could rescue gene expression in cardiac pMFBs from WT and KO mice via adenoviral transgene delivery. KO cells exhibited virtually no Scx expression, but AdScx drove Scx to approximately sixfold of WT expression (Fig. 6a). The significant loss of *Vim*, *Acta2*, *Fn1*, *Col1a1*, *Col1a2* and *Col3a1* observed in these cells was similarly rescued (Fig. 6a). Loss of expression of these genes in Scx null cells is thus not due to a generalized defect in the transcription mechanism, but rather is specific to loss of Scx transcriptional activity. In accordance with our earlier data, neither loss nor rescue of Scx altered *Col4a1* expression.

We previously demonstrated strong synergy between Scx and Smad3 in regulating *Col1a2* expression, and that deletion of Scx-binding sites in the *Col1a2* promoter attenuated Smad3 transactivation of this promoter [18]. We therefore examined the impact of Scx knockdown on Smad3-mediated collagen gene expression in primary pMFBs. Smad3 induced expression of the fibrillar collagens *Col1a1*, *Col1a2* and *Col3a1*, but this was significantly attenuated by Scx knockdown (Fig. 6b). Given this dependence of Smad3 activity on Scx, we tested whether Smad3 and Scx physically interact using co-immunoprecipitation of Scx and Smad3. We observed a clear interaction between the two proteins (Fig. 6c), in agreement with a recent report that Smad3 binds Scx in tendons [27]. Deletion of the helix-loop-helix protein interaction domain of Scx attenuated binding to Smad3.

Given the close physical and functional relationship between Scx and Smad3, we examined the effect of Scx loss on Smad3 recruitment to the *Col1a2* gene promoter by chromatin immunoprecipitation. Smad3 binding to the *Col1a2* gene promoter Smad binding element was significantly reduced in Scx KO hearts compared to WT (Fig. 6d), and re-ChIP demonstrated a similar loss of Smad3-bound RNA polymerase II. This effect was recapitulated in a dose-dependent manner by delivering a Scx dominant-negative mutant ScxΔBD [18] to human adult ventricular MFBs (Fig. 6e), suggesting that the mutant exerts its effect by sequestering Smad3 away from the promoter. Together, these results demonstrate that Smad3 signaling is attenuated in the absence of Scx due to impaired transcriptional complex formation.

### Loss of cardiac fibroblasts in scleraxis null mice

**Fig. 7:**
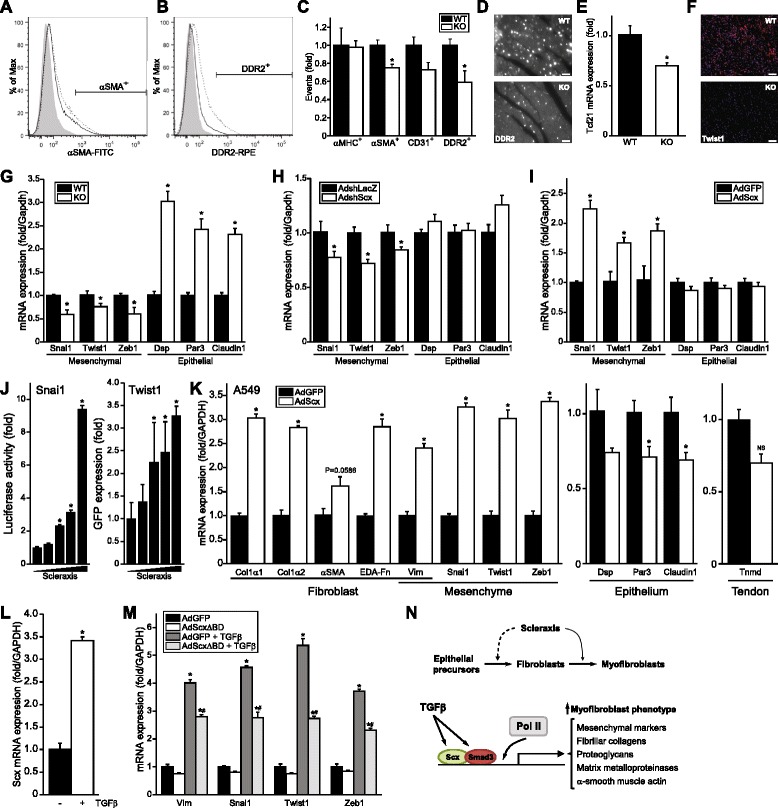
Loss of cardiac fibroblasts following scleraxis deletion *in vivo*. **a**–**b** Representative flow cytometry histograms of WT and Scx KO heart single-cell suspensions show cardiac cells stained for αSMA (**a**) or DDR2 (**b**) in WT (*dotted line*) and KO (*solid line*) mice. Unstained cell sample was used as control (*shaded histogram*). **c** Total αMHC^+^, αSMA^+^, CD31^+^ or DDR2^+^ stained events per 5 × 10^4^ events in WT or KO mice normalized to WT counts demonstrating normal cardiomyocyte counts but reduced numbers of fibroblasts; *n* = 3 independent samples per genotype. **d** Representative cardiac sections from WT and KO mice immunolabeled for DDR2 confirm the loss of DDR2^+^ cells; 63× objective, scale bar = 20 μm. **e** qPCR assay of Tcf21 mRNA expression in WT and Scx KO mice; *n* = 3 independent samples per genotype. **f** Cardiac sections as in (**d**) were immunolabeled for Twist1 expression (*red*; DAPI nuclear staining in *blue*); 20× objective, scale bar = 64 μm. **g** Cardiac mRNA from WT and Scx KO mice was assayed for expression of mesenchymal and epithelial marker genes by qPCR, indicating reduced epithelial-to-mesenchymal transition in KO hearts; *n* = 5 independent samples per genotype. **h**–**i** Cardiac proto-myofibroblasts were subjected to Scx knockdown (**h**) or over-expression (**i**) and EMT markers assessed by qPCR, indicating that Scx regulates mesenchymal marker gene expression; *n* = 3. **j** Scx dose-dependently transactivates the Snai1 and Twist1 proximal gene promoters as determined by luciferase assay or GFP western blot, respectively; *n* = 3. **k** Representative fibroblast/myofibroblast, mesenchymal, epithelial and tendon marker gene mRNA expression was assayed in A549 epithelial cells following Scx over-expression (AdScx) vs controls (AdGFP), assayed by qPCR; *n* = 3. **l** Scx mRNA expression was assayed by qPCR in A549 cells following treatment with 2.5 ng/mL TGFβ or vehicle; *n* = 4. **m** Mesenchymal marker gene mRNA expression was assayed in A549 cells following Scx dominant negative mutant over-expression (AdScxΔBD) vs controls (AdGFP), with or without treatment with 2.5 ng/mL TGFβ or vehicle; *n* = 3. *P < 0.05 vs WT (**c**, **e**, **g**), vs AdshLacZ (**h**), vs AdGFP (**i**, **k**, **m**), vs control transfected vector (**j**) or vs vehicle (**l**); #*P* <0.05 vs AdGFP + TGFβ (**m**). **n** Putative model of action of Scx. Top panel, Scx is required for cardiac fibroblast to myofibroblast phenotype conversion (*solid arrow*), and possibly for transition of epithelial precursors to the mesenchymal/fibroblast phenotype (*dashed arrow*). *Bottom panel*, Scx is sufficient to directly transactivate numerous genes that characterize the myofibroblast phenotype, and is required for TGFβ/Smad3-mediated gene expression by facilitating Smad3 and RNA polymerase II interaction at target gene promoters such as *Col1α2*

Scx loss clearly impairs cardiac FB function, but it is also possible that cardiac FB number may be negatively impacted. We analyzed cardiac cell identity by flow cytometry using markers for cardiomyocytes (α-myosin heavy chain, αMHC), FBs (DDR2), vascular smooth muscle (αSMA) and endothelial cells (CD31) (Additional file 3: Figure S2). The number of αSMA^+^ and DDR2^+^ cells was significantly reduced with a similar trend in CD31^+^ cells (Fig. 7a–c). Since αSMA is primarily expressed by vascular smooth muscle cells in the healthy heart, the cardiac vasculature may be negatively impacted in these animals. A significant ~25 % decrease in capillary density in KO hearts further indicates reduced vascularity (Additional file 4: Figure S3A–B). Although αMHC^+^ cardiomyocyte number was not affected by Scx knockout, we found that cardiomyocyte cross-sectional area significantly decreased by 29 % in KO mice (Additional file 4: Figure S3C–D). The net hypotrophy noted in KO hearts thus appears to be due to reduced myocyte volume.

DDR2 is enriched in cardiac FBs and not expressed in cardiomyocytes [28, 29]. Nearly half of all DDR2^+^ cells were lost in Scx KO hearts (Fig. 7b–c), and cardiac tissue sections exhibited greatly reduced numbers of DDR2-expressing FBs (Fig. 7d). It is unlikely that cell loss is due to increased cell death, as shRNA-mediated knockdown of Scx had no such effect (Additional file 5: Figure S4). The loss of cardiac fibroblasts may be responsible for the decrease in cardiomyocyte size, since co-culture of fibroblasts with adult cardiomyocytes promotes hypertrophy [30].

We hypothesized that missing DDR2^+^ cells in KO hearts may reflect an altered cell fate of FB precursors, rather than actual cell loss. Differentiation of cardiac FBs during development, with further phenotype refinement to MFBs, has been ascribed to epithelial-to-mesenchymal transition (EMT) [31, 32]. In KO hearts, we observed a ~30 % loss of mRNA expression of Tcf21 (Fig. 7e), a transcription factor required for EMT of cardiac FBs from proepicardial organ precursors [31], thus we assayed EMT marker expression using a selection of epithelial and mesenchymal marker genes that, while not individually definitive for EMT, have collectively been widely used in the literature to assess EMT progression. Immunofluorescence revealed a notable loss of labeling for the mesenchymal marker *Twist1* in KO hearts, with fewer *Twist1*-positive nuclei consistent with a loss of fibroblasts (Fig. 7f). mRNA of all mesenchymal markers tested (*Snai1*, *Twist1*, *Zeb1*) was significantly down-regulated by 25–40 % in KO hearts, while epithelial markers (*Dsp*, *Par3*, *Cldn1*) increased (Fig. 7g). While a definitive assessment of embryonic EMT is beyond the scope of the present study, this result is consistent with an attenuation of EMT during the developmental process. Knockdown of Scx in pMFBs significantly reduced mesenchymal marker expression, while Scx over-expression had the opposite effect (Fig. 7h, i). Scx dose-dependently transactivated the *Snai1* and *Twist1* gene promoters (Fig. 7j).

Strikingly, over-expression of Scx in the A549 human lung epithelial cell line caused a potent up-regulation of a variety of FB and mesenchymal markers while reducing epithelial marker expression (Fig. 7k), demonstrating that Scx was sufficient to induce EMT in these cells. Furthermore, Scx did not alter expression of the tendon marker *Tnmd* in A549 cells, indicating that Scx induced a FB rather than a tenocyte phenotype. This result is in contrast to C3H10T1/2 mouse embryonic FBs, in which Scx induced FB and mesenchymal markers as well as *Tnmd* (Additional file 6: Figure S5), in agreement with a recent report that Scx induces a tenocyte fate in these cells [33]. We note that induction of FB and mesenchymal markers in either cardiac pMFBs or C3H10T1/2 cells required adenoviral gene delivery of Scx at only 10 MOI—an order of magnitude less than the amount required to induce EMT in A549 cells (100 MOI). The ability of Scx to regulate a mesenchymal FB phenotype thus appears to be cell type-specific and dose-dependent. TGFβ induces EMT in A549 cells [34], and we observed potent up-regulation of Scx by TGFβ similar to our findings in cardiac FB (Fig. 7l) [18]. The induction of mesenchymal marker expression by TGFβ was significantly attenuated by our Scx dominant-negative mutant ScxΔBD, demonstrating a requirement for Scx in this process (Fig. 7m).

## Discussion

The transcriptional regulators that govern FB phenotype and function are poorly defined. Our study demonstrates for the first time that Scx is a critical regulator of virtually all hallmarks of the mesenchymal phenotype of FBs and MFBs. This includes both sufficiency and necessity for the *ex vivo* and *in vivo* expression of numerous ECM genes, FB/MFB markers, αSMA and cell contraction, and mesenchymal markers—expression patterns that virtually define the FB and MFB phenotypes. We also provide the first evidence that Scx could potentially be important for EMT programming of FBs in the myocardium.

Tendons and cardiac valves possess a collagen-rich ECM, and Scx KO profoundly affects both tissues. Scx deletion results in the loss of force-transmitting tendons due to a failure of tendon progenitor differentiation, disorganization of the tendon matrix and loss of type I collagen production via an undefined mechanism [17]. Conversely, Scx commits embryonic stem cells to a tenocyte fate and induces expression of *Tnmd*, a direct Scx target gene in C3H10T1/2 cells [33, 35, 36]. Scx KO mice exhibit thickened cardiac valves with altered collagen expression and fiber organization due to reduced valve precursor cell differentiation, although Scx target gene identity was unclear [15]. Scx thus appears to regulate progenitor cell behavior and tissue structure in ECM-rich environments through undefined mechanisms, resulting in significant cell fate and ECM alterations when this function is disturbed.

Our results indicate that Scx performs a fundamental role in FB cell fate/phenotype in the collagen-rich myocardium, and for the first time reveal the underlying mechanisms. Our identification of *Vim*, *MMP2*, *Fn1*, *Acta2*, *Snai1* and *Twist1* as novel transcriptional targets of Scx (Figs. 3d, 4h–k, 7j) explains the loss of expression of these genes following Scx loss, and complements previous work by our laboratory and others identifying the *Col1a1* and *Col1a2* genes as additional direct targets [6, 18, 19]. We anticipate that other genes assayed here, including *Col3a1*, *Col5a1*, *Fmod*, *Lum*, *DDR2*, *Myh10* and *Postn*, are likely to also be direct transcriptional targets given the close agreement between their expression and Scx. However, our work also reveals the critical interplay between Scx and Smad3, including physical interaction (Fig. 6b–c), resulting in impaired assembly of a transcriptional complex at the *Col1a2* promoter in Scx null mice or at the *COL1A2* promoter following treatment of human adult ventricular FBs with our dominant-negative Scx mutant (Fig. 6d–e), as well as complete attenuation of TGFβ-mediated pMFB contraction (Fig. 4g). The dominant-negative mutant lacks a DNA-binding domain, and we had hypothesized that this mutant acted by sequestering key binding partners away from target genes [18]. Here we demonstrate that this mutant dose-dependently reduced Smad3-binding to the COL1A2 promoter, in turn reducing recruitment of RNA polymerase II (Fig. 6e), in support of this model. Together, these results provide the mechanism for our previous observation of both the synergy between Smad3 and Scx, as well as their mutual requirement for full transcriptional activity on the *COL1A2* promoter [18], and suggests a model for how Scx may interact with Smad3 or other binding partners to modulate additional downstream genes.

In both tendons and cardiac valves, Scx gene deletion resulted in a failure of progenitor cells to specify, differentiate and/or proliferate, but without apoptosis [15, 17]. It was also recently shown that Scx is required for tendon stem cell differentiation to tenocytes [37]. Our data fits with the theme of Scx behaving as a cell fate mediator. EMT plays an important role in the generation of FBs in the adult myocardium [31, 32], and we show that Scx directly transactivates the gene promoters of the mesenchymal markers *Vim*, *Snai1* and *Twist1* (Figs. 3d, 7j). The loss of mesenchymal marker expression in Scx KO hearts, with increased epithelial marker expression, supports the possibility that Scx may be important in the developmental EMT programming of FBs from epithelial precursors (Fig. 7n), and we show for the first time that Scx is sufficient to reprogram A549 epithelial cells to a mesenchymal, FB-like phenotype (Fig. 7k). TGFβ/Smad3 signaling is a potent driver of EMT [38]. We found that TGFβ up-regulated Scx expression in A549 cells, and ScxΔBD significantly attenuated TGFβ-mediated mesenchymal marker induction (Fig. 7k, m), demonstrating a requirement for Scx in this process and consistent with a central role for scleraxis in mediating TGFβ/Smad3 downstream events. The loss of FBs in Scx KO hearts may thus reflect a failure of EMT. The regulation of EMT in the embryonic myocardium by Scx is an intriguing focus for future study, requiring direct assessment of EMT and nascent FB numbers during development. FB to MFB conversion is an extension of this EMT, with further refinement of the cell phenotype to increase ECM synthesis, induce expression of αSMA and other MFB genes, and initiate cell contraction. Here, too, Scx was both necessary and sufficient to govern broad ECM gene expression (Figs. 1, 2, 5), to directly transactivate the αSMA promoter (Fig. 4i–k), to facilitate cell contraction (Fig. 4e–g) and to regulate FB/MFB marker gene expression (Figs. 3, 5h–i). Scx thus governs the main features of fibroblastic cells in the heart along the full phenotype continuum.

In the developing mouse heart, high pericardial expression of scleraxis was observed at embryonic day 11 [39]. This is consistent with the localization of proepicardial organ-derived Scx^+^ cells at the epicardial surface at the same time point reported by Katz et al. [40]. However, Katz also noted that Scx^+^ cells had already migrated into the right ventricle by E10.5, suggesting they had undergone a prior EMT event, and migration continued to at least E13.5. Approximately two thirds of WT1^+^ cells in the Katz study did not express Scx, and of the Scx^+^ cells, 60 % gave rise to fibroblasts, epicardium and other derivatives, 24 % gave rise to endothelial cells, 7.8 % gave rise to smooth muscle cells, and 6.6 % gave rise to cardiomyocytes. Thus it appears that a sub-population of cells expressing Scx gives rise to FBs, although the exact size of this cell population remains unclear. However, the existence of such a sub-population could explain why Scx KO hearts lacked only ~50 % of FBs, rather than exhibiting the nearly complete loss that was observed in Tcf21 null hearts [31]. Tcf21 KO and Scx KO hearts bear a striking physical resemblance to one another, including right-side enlargement, and rounding and involution of the apex, and Tcf21 has been shown to regulate Scx expression in Sertoli cells [15, 31, 41]. Scx KO may thus partially phenocopy Tcf21 loss, and investigation of a similar regulatory regime in the myocardium is warranted. In this regard, it is noteworthy that Tcf21 was down-regulated in Scx KO hearts (Fig. 7e). An intriguing possibility is that a net loss of fibrillar collagen and extracellular matrix, and thus structural support, in the thinner and physically weaker right ventricular wall may lead to specific remodeling of this chamber, consistent with the right ventricular enlargement observed in both Scx KO and Tcf21 KO hearts. It is also notable that, unlike Tcf21 KO hearts, we observed evidence of reduced smooth muscle and endothelial cell numbers. This is consistent with Katz’ report of Scx^+^ cells contributing to these cell populations, and may reflect an impairment in the development of these populations. Katz also noted that Scx^+^ cells contributed in a minor fashion to the cardiomyocyte population. While we saw no evidence of a decline in cardiomyocyte number, we did note a reduction in cardiomyocyte size (Additional file 4: Figure S3C–D), suggesting that Scx may play a role in the growth of these cells. However, we cannot rule out the possibility that this alteration may instead reflect a response of cardiomyocytes to the altered extracellular matrix environment present in Scx KO hearts.

The FBs that do arise in the Scx KO heart appear to be functionally impaired: pMFBs isolated from KO hearts exhibited dramatic loss of *Col1a1*, *Col1a2*, *Col3a1*, *Vim*, *Fn1* and *Acta2*, demonstrating a clear functional deficit in these cells (Fig. 6a). However, this deficit could be corrected simply by restoring Scx expression, further demonstrating the critical central role of Scx in fibroblastic gene expression. Furthermore, this effect was dependent on intact DNA-binding and protein-interaction moieties within Scx, as a mutant lacking these sequences failed to impact gene expression (Additional file 1: Figure S1).

Transition of FBs to MFBs in connective tissues of the heart, lungs, liver, kidney and dermis is integral to pathologic ECM remodeling and scar formation [42]. While the identity of definitive FB/MFB markers remains controversial, Scx induced and was required for the expression of virtually all commonly-employed FB/MFB markers, including *DDR2*, *Fn1*, *Myh10*, *Vim* and *Acta2* (Figs. 3, 4 and 5) [3, 12, 28, 29]. Scx also induced the incorporation of αSMA into stress fibers (Fig. 4d), a hallmark of full conversion to MFBs [16], and we found that Scx potently up-regulated *Postn* (Fig. 3a), which is implicated in cardiac fibrosis [23, 43, 44]. Promotion of the MFB cell fate and induction of ECM synthesis by Scx is clearly congruent with a role in cardiac fibrosis.

Further investigation into the specific role of Scx in cardiac fibrosis and ECM remodeling is thus an important next step, particularly given our finding that pro-fibrotic TGFβ/Smad3 signaling potently up-regulate Scx expression [18]. Investigation of the impact of Scx loss on cardiac fibrosis is beyond the scope of the present inquiry, but the potential for Scx to play a pathogenic role is clear. The cardiac developmental defects noted in Scx KO mice, including abnormal valve formation, prevents their use in experimental models of cardiac dysfunction such as pressure overload, infarction or β-agonist administration, but conditional gene deletion offers an alternative strategy to address this important question in future studies. The central role of Scx in FB phenotype and ECM synthesis demonstrated here should spur investigation into methods of attenuating Scx function in order to develop novel therapeutic strategies for fibrosis, which at present are completely lacking [45].

## Conclusions

The results presented here identify the transcription factor scleraxis as not only a potent activator of ECM gene expression and synthesis *in vitro* and *in vivo*, but also and for the first time as a key regulator of the phenotype of cardiac fibroblasts. Epithelial cells, mesenchymal fibroblasts and myofibroblasts represent a cellular continuum from development to disease, and our data demonstrate the central role of scleraxis in the transition of cells through these phenotypes. Although a definitive role for scleraxis in EMT during fibroblast formation in cardiac development remains to be demonstrated, scleraxis induced and was required for EMT in A549 epithelial cells, and the loss of fibroblasts concomitant with up-regulation of epithelial markers (and loss of mesenchymal markers) in the scleraxis null heart is consistent with a role in developmental EMT. Fibroblast-to-myofibroblast transition was attenuated by scleraxis gene deletion or knockdown, and was induced by scleraxis over-expression. Given the significant contributory role of fibroblast activation in fibrotic diseases, and the conservation of key signaling pathways, including TGFβ/Smad in fibrosis across tissue types, scleraxis represents an attractive new target for the development of therapies targeting fibrosis in the heart and other tissues.

## Methods

### Animal studies

The generation of scleraxis null mice (strain Scx^tm1.1Stzr^; MGI:3716564) has been described previously [17]. Mice were on a C57BL/6 background, and wild type littermates were used as controls. Animals were provided with food and water *ad libitum* and maintained on a 12/12 day/night cycle. All animals were cared for as prescribed by the guidelines of the Canadian Council on Animal Care (CCAC) and the University of Manitoba Animal Care Committee. Wild type and knockout animals were analyzed at 6–10 weeks of age. For analyses of gene expression in wild type and scleraxis null hearts, ventricular myocardium was dissected clear of the atria and valves.

### Antibodies

Antibodies used throughout the study are listed in Additional file 2: Table S2.

### Echocardiography

Non-invasive murine transthoracic echocardiography was performed on 6-week-old, non-sedated animals using a Vivid 7 machine (GE Healthcare), equipped with a 13 MHz linear probe [46, 47]. Cardiac dimensions and function were evaluated using 2D and M-mode data collected in long and short parasternal axis views, as well as tissue Doppler imaging. Data collection and analysis were performed separately by observers blinded to the genotype of the animals.

### Ventricular ECM mass determination

Cardiac ventricles from WT or scleraxis KO animals were removed and fragmented. Tissue fragments were processed for decellularization using a conventional cell maceration technique with modifications [48, 49]. Briefly, tissues were fixed in 10 % neutral buffered formalin. After 4–5 thorough rinses in distilled water, the tissue fragments were immersed in a 10 % sodium hydroxide solution for 4–5 days to remove the cellular elements. The decellularized tissue was rinsed with water until it became transparent, then dehydrated in 100 % methanol followed by freeze-drying to yield dry ECM, and the dry mass recorded.

### Cell culture and treatments

Primary cardiac fibroblasts were isolated from adult male Sprague Dawley rats via Langendorff perfusion and enzymatic digestion using 0.1 % collagenase type II (Worthington Biochemical Corporation) followed by gentle centrifugation for collection of cells as previously described [18, 50]. Cells were maintained in DME/F12 media supplemented with 10 % serum, 1 % antibiotics and 1 mmol/L ascorbic acid. Cells were then passaged once to P1 (passage 1) proto-myofibroblasts and plated appropriately for downstream assays [6]. Equilibration of cells for 24 h in serum-free medium was carried out prior to all treatments. For over-expression studies, cells were infected with adenoviruses encoding LacZ (AdLacZ), GFP (AdGFP) or scleraxis (AdScx) for 24 h; we have previously shown that AdScx induces physiologic levels of scleraxis expression (by approximately threefold) similar to treatment with TGFβ [6, 18]. For experiments requiring treatment of cells with TGF-β_1_, media was replenished and cells treated for an additional 24 h with recombinant TGF-β_1_ or vehicle (control). The ScxΔΔ mutant was generated using nested PCR based on our previous mutant [6]. Primary rat cardiac proto-myofibroblasts (5 × 10^5^ cells) were transfected by electroporation (Gene Pulser II, Bio-Rad) at 220 V and 500 μF in 0.5 mL serum-supplemented DME/F12 media with 2 μg pECE (control) or ScxΔΔ. Total RNA was isolated from these cells 24 h post-transfection and analyzed for target gene expression. For some studies, cardiac fibroblasts were isolated from 6-week-old WT and scleraxis KO mice using Liberase TH Research Grade (Roche) [51]. Cells from the first passage were used for adenovirus-mediated gene rescue experiments. A549 human alveolar epithelial carcinoma cells (ATCC) were maintained in DMEM high glucose media supplemented with 10 % FBS and 1 % antibiotics. Cells were incubated overnight in 1 % insulin-transferrin-selenium (ITS; Gibco)-supplemented medium and infected with adenovirus encoding scleraxis or GFP (control). C3H10T1/2 mouse embryonic fibroblasts (ATCC) were cultured and treated similarly as A549 cells. Total RNA was isolated from these cells and subjected to qPCR using specific primers.

### Generation of shRNA-encoding adenovirus

The BLOCK-iT RNAi Advisor program (Life Technologies) was used to design shRNA sequences for rat scleraxis gene knockdown. The two pairs of oligonucleotides were annealed and cloned into the pENTR/U6 RNAi Entry Vector following the manufacturer’s instructions (Life Technologies), then subcloned into pAd/BLOCK-iT-DEST vector to generate pAd-shScleraxis. The vectors were finally packaged to produce Ad-shScleraxis adenovirus (AdshScx) in 293A cells. Viral titer was determined using a commercial kit (Adeno-X Rapid Titer Kit) following manufacturer’s instructions (Clontech Laboratories, Inc.). P1 rat cardiac proto-myofibroblasts were infected at various multiplicities of infection (MOI; 50, 100, 200) for 48 h to determine optimal MOI for further experiments. All experiments were carried out using MOI 200 for 72 h. Infection control groups received adenovirus encoding shLacZ (AdshLacZ) [52]. The following oligonucleotides were used for scleraxis knockdown: 5′-CACCGCCTCAGCAACCAGAGAAAGTCGAAACTTTCTCTGGTTGCTGAGGC-3′ (forward) and 5′-AAGCCTCAGCAACCAGAGAAAGTTTCGACTTTCTCTGGTTGCTGAGGC-3′ (reverse).

### Cell viability

Primary adult rat cardiac fibroblasts were grown in Permanox plastic chamber slides (Nunc, Thermo Fisher Scientific) in DME/F12 medium (HyClone, GE Healthcare) supplemented with 0.5 % FBS and 500 μmol/L ascorbic acid. Cells were infected with AdshLacZ (control) or AdshScx adenovirus at MOI 200 for 48 h. Cell viability was assayed using the LIVE/DEAD Viability/Cytotoxicity kit as per manufacturer’s instructions (Life Technologies). Vital dyes calcein acetoxymethyl ester (calcein AM) and ethidium homodimer-1 were used to visualize live (green) and dead (red) cells, respectively, using a Zeiss Axio Imager M1 epifluorescence microscope with 10× EC Plan-Neofluar objective (NA 0.3). Data was recorded as percent cell death compared to control from three independent experiments (minimum total cell count of 400); 200 μmol/L H_2_O_2_-treated cells were used as dead cell control.

### Quantitative real-time PCR (qPCR)

Total RNA was collected from isolated cells using a kit as per manufacturer’s directions (Thermo Fisher Scientific). Cardiac tissue RNA was isolated using TRIzol Reagent (Ambion, Life Technologies) as per manufacturer’s instructions. Equal amounts of RNA (25 ng for cells, 50 ng for tissue) were assayed using an iQ5 multicolor real-time PCR thermocycler (Bio-Rad) using the qScript One-Step SYBR Green qRT-PCR kit for iQ (Quanta Biosciences). Amplicon abundance was quantified using the 2^-ΔΔCT^ method, and normalized to *Gapdh*. Primers used for these reactions are per our previous work or listed in Additional file 2: Table S3 [6, 18].

### Western blotting

Total protein was isolated from cells using radioimmunoprecipitation assay buffer (RIPA) comprised of 50 mmol/L Tris pH 7.4, 150 mmol/L NaCl, 1 mmol/L EDTA, 1 mmol/L EGTA, 0.5 % Na-deoxycholate, 1 % Triton X-100 and 0.1 % sodium dodecyl sulfate (SDS). Protease inhibitor cocktail (Thermo Fisher Scientific), 1 mmol/L DTT and 1 mmol/L PMSF were added to the RIPA buffer prior to use. Mouse tissue lysates were prepared by homogenizing cardiac tissue in protein extraction reagent type 4 (Sigma-Aldrich). Proteins were separated under reducing conditions using SDS polyacrylamide gel electrophoresis and transferred onto polyvinylidene difluoride membranes (Pall Corporation). Blots were probed with specific primary antibodies for scleraxis [6, 18]: collagen type I (Cedarlane); collagen type III, type IV or α-tubulin (Developmental Studies Hybridoma Bank, University of Iowa); Gapdh, DDR2 or αSMA (Sigma-Aldrich); vimentin or SM_emb_ (Abcam); and ED-A fibronectin (Millipore) or β-actin (Cell Signaling Technology). Blots were then incubated with appropriate HRP-conjugated mouse or rabbit secondary antibodies. Protein bands were visualized using Western Blotting Luminol Reagent (Santa Cruz Biotechnology) on CL-Xposure blue X-ray film (Thermo Fisher Scientific). Band intensities were quantified using Quantity One (Bio-Rad) or ImageJ (National Institutes of Health; NIH) applications. Target band intensity was normalized to Gapdh, β-actin or α-tubulin.

### Gelatin zymography

Serum-free conditioned cell culture medium from P1 proto-myofibroblasts was collected at 24 h or 48 h post-infection with AdGFP or AdScx, and concentrated using centrifugal filter devices (Millipore). Cardiac tissue extracts from WT and KO mice were prepared using lysis buffer (25 mmol/L Tris-HCl pH 7.5, 100 mmol/L NaCl, 1 % NP-40) supplemented with protease inhibitors [53]. Next, 20 μg concentrated proteins (culture media or tissue lysate) was electrophoresed under denaturing conditions using commercial pre-cast 10 % acrylamide gels containing 0.1 % gelatin (Novex, Life Technologies). Gels were then renatured in 2.5 % Triton X-100 for 30 min at room temperature followed by incubation in developing buffer (50 mmol/L Tris base, 40 mmol/L HCl, 200 mmol/L NaCl, 5 mmol/L CaCl_2_ and 0.02 % Brij 35) at 37 °C for 16 h in a closed tray. Gelatinolytic activity was then visualized by staining with Coomassie Brilliant Blue R-250 dye (0.5 % in 5 % methanol and 10 % acetic acid) followed by destaining (10 % methanol, 5 % acetic acid) to reveal clear bands over the blue background of the dye. Gels were dried and scanned on a densitometer, and band intensities were quantified using Image J software (NIH) [54].

### Flow cytometry

Whole hearts below the atria were isolated from WT and scleraxis KO mice, and were immediately flushed with ice-cold HBSS. The method described by Banerjee et al. was used with modifications [55]. Briefly, hearts were immersed in HBSS supplemented with 1 mg/mL bovine serum albumin (BSA), minced and repeatedly passed through a 19-gauge needle, then incubated for 5 min with shaking at 100 rpm at 37 °C. The supernatant was collected, and the rest of the tissue was subjected to enzymatic digestion using 140 U/mL collagenase supplemented with 10 mg/mL BSA. This process was repeated six times at 37 °C. The supernatant was pooled from all steps and centrifuged at 1,000 × *g* for 5 min at 4 °C. The supernatant was discarded and cells resuspended in Gey’s solution for 5 min on ice to lyse red blood cells. After centrifugation, cells were resuspended in 1 mL staining buffer (phosphate-buffered saline with 1 % FBS). Cells were then counted and resuspended at 0.5 million cells/100 μL for flow cytometry analysis. Cells were processed using the BD Cytofix/Cytoperm kit (BD Biosciences) as per manufacturer’s instructions. Single-cell suspensions from both WT and KO groups (*n* = 3) were stained with either FITC-conjugated-αSMA (Abcam), R-PE-conjugated-DDR2, APC/Cy7-conjugated αMHC or PE/Cy7-conjugated-CD31 (BD Biosciences) antibodies. Fluorochrome conjugation for DDR2 and αMHC was performed using EasyLink Kits (Abcam). Acquisition of cell samples was performed on a FACSCanto II cytometer using FACSDiva software (BD Biosciences). Data were analyzed with FlowJo software (Tree Star, Inc.).

### Histology

Cardiac tissues from KO mice or WT littermates were formaldehyde-fixed, embedded in OCT or paraffin and processed for assessment of histological features. Next, 5 μm sections were subjected to Masson’s trichrome staining using commercial reagents (Sigma-Aldrich). Briefly, tissue sections were mordant in pre-heated Bouin’s solution (Sigma-Aldrich) for 15 min at 56 °C, then rinsed with tap water to remove excess reagent. Sections were stained with Weigert’s hematoxylin solution (Sigma-Aldrich), rinsed sequentially in tap and deionized water, and then stained with the trichrome kit. Slides were treated with 1 % acetic acid, dehydrated through alcohol, cleared in xylene and mounted using Permount (Thermo Fisher Scientific). For discrete visualization of collagen deposits in the myocardium, tissue sections were stained with picrosirius red (PSR). Slides were incubated in Bouin’s solution as above, followed by staining in picrosirius red (0.1 %) for 60 min at room temperature. Sections were then washed with 1 % acetic acid twice, dehydrated, cleared and mounted using Permount. Both trichrome and PSR-stained sections were examined using a Zeiss Axioskop 2 mot plus microscope equipped with an AxioCam digital camera, 40× Plan-Neofluar objective (NA 0.75) and AxioVision 4.6 software (Zeiss). Alternatively, PSR-stained sections were visualized on an Axio Imager M1 microscope equipped with a circular polarizer filter (Zeiss), AxioCam HR digital camera and 40× objective as above. For enhanced visualization of blue staining in Masson’s trichrome sections, images were processed with Adobe Photoshop CS6 to remove the red and green color channels.

### Luciferase reporter assay

NIH-3T3 cells were plated in 6-well dishes and transfected at ~70 % confluence. After equilibration in Opti-MEM (Gibco) for 30 min, cells were co-transfected (Lipofectamine 2000, Life Technologies) with one of the following luciferase reporter vectors: rat pGL3-αSMA [56] (courtesy Dr Sem H. Phan, University of Michigan), human pGL2-MMP2 [57] (courtesy Dr Etty Benveniste, University of Alabama), human pGL3-vimentin [58, 59] (courtesy Dr Christine Gilles, University of Liege) or human pGL3-fibronectin [60] (courtesy Dr Jesse Roman, University of Louisville), and pECE (control) or scleraxis expression vectors [6, 18] for 24 h. Renilla luciferase expression vector (pRL) was used for normalization of luciferase reporter activity. Luciferase activity in the samples was analyzed using the Dual-Luciferase Reporter Assay System (Promega) on a Glomax-Multi + Microplate Multimode Reader (Promega) equipped with Instinct software. Point mutations were introduced in the E-box sequences of the 0.7 kb αSMA promoter construct using site-directed mutagenesis (Agilent Technologies) (Additional file 2: Table S4) and verified by sequencing. A scleraxis double deletion mutant (ScxΔΔ) was generated using nested PCR (Additional file 2: Table S5).

### Electrophoretic mobility shift assay

Gel mobility shift assays were performed as described previously [18]. Biotin-labeled oligonucleotides and cold probes (Additional file 2: Table S6) were synthesized commercially (Integrated DNA Technologies). The assays were performed using a LightShift Chemiluminescent EMSA Kit (Pierce Biotechnology) according to manufacturer’s instructions.

### Chromatin immunoprecipitation (ChIP/re-ChIP) assay

Assays were performed as described previously [18]. Solubilized chromatin from adult rat cardiac fibroblasts and myofibroblasts was incubated overnight at 4 °C with 10 μg anti-scleraxis antibody or pre-immune serum. Protein-DNA complexes were isolated from scleraxis WT and KO cardiac tissues using a commercial kit (Millipore) and incubated with 5 μg anti-Smad3 antibody (Abcam) or rabbit IgG (Santa Cruz Biotechnology) overnight at 4 °C. For re-ChIP assays with anti-RNA Polymerase II Antibody (Millipore), after the elution of the first ChIP assay, the samples were diluted ten times with re-ChIP dilution buffer (15 mmol/L Tris-HCl pH 8.1, 1 % Triton X-100, 1 mmol/L EDTA, 150 mmol/L NaCl) and subjected to the ChIP procedure again. Negative controls included performing re-ChIP assays with mouse IgG (Millipore). The immunoprecipitates were collected and eluted DNA was subjected to qPCR using specific primers (Integrated DNA Technologies; Additional file 2: Table S7) [61] and SYBR Green reagents (Quanta Biosciences). Fold enrichment was calculated (ΔC_t_) and represents the ratio of protein-bound DNA (scleraxis, Smad3 or RNA Pol II) to a negative control (pre-immune serum or non-specific IgG) normalized for input DNA. A region of *Gapdh* promoter (rat or mouse) was amplified to represent negative control for the experiments; no differences were observed for scleraxis or Smad3-binding to the *Gapdh* promoter (*P* > 0.05; *n* = 3). For experiments using dominant negative scleraxis mutant, human cardiac myofibroblasts were infected with adenoviruses encoding the mutant (AdScxΔBD) or GFP (AdGFP; control) at MOIs for 24 h followed by ChIP and re-ChIP procedures as mentioned above.

### Co-immunoprecipitation (Co-IP)

NIH-3T3 cells were transfected with the expression vectors encoding scleraxis (pECE-HA-Scx), scleraxis protein-binding domain mutant (pECE-HA-ScxΔHLH), Smad3 (pCMV tag3C-Myc-Smad3; courtesy Dr Jean-Jacques Lebrun, McGill University) or combinations using Lipofectamine 3000 (Life Technologies) for 48 h. Cells were then rinsed once with PBS and lysed in 50 mmol/L Tris (pH 7.5), 150 mmol/L NaCl and Triton X-100 (0.5 %). Cell membranes were ruptured by passing the lysate through a syringe with a 25-gauge needle. Total protein was collected after centrifugation of lysates at 14,000 rpm at 4 °C for 20 min. Protein complexes (500 μg) were immunoprecipitated using an anti-HA antibody (Rockland Immunochemicals Inc.) or rabbit IgG (Santa Cruz Biotechnology), resolved on a 12 % polyacrylamide gel followed by western blotting with an anti-c-myc antibody (Developmental Studies Hybridoma Bank). Whole-cell extracts were used for immunoblotting with anti-HA or anti-c-myc antibody to confirm transfection of expression vectors.

### Immunofluorescence imaging

Cardiac P1 proto-myofibroblasts were plated onto glass coverslips infected at MOI 10 with AdLacZ or AdScx for 24 h. Cells were then fixed in 2 % paraformaldehyde (Alfa Aesar), permeabilized in 0.3 % Triton X-100 and 0.1 % Na-citrate, and blocked in 5 % goat serum (Life Technologies). Cells were incubated with FITC-αSMA antibody (Abcam) at 4 °C overnight followed by mounting onto slides using SlowFade Gold Antifade reagent with DAPI (Molecular Probes, Thermo Fisher Scientific). For DDR2, collagen I and Twist1 staining, paraffin-embedded 5 μm tissue sections were deparaffinized and rehydrated to distilled water. Tissue slides were processed for antigen retrieval using a citrate-based commercial reagent (Vector Laboratories) at 90 °C for 30 min and then cooled at room temperature for another 30 min. Sections were then washed with PBS and incubated in blocking reagent (Roche Applied Science) for 1 h at room temperature. Slides were incubated with antibodies against DDR2 (Sigma-Aldrich), collagen I (Cedarlane) or Twist1 (Sigma-Aldrich) at 4 °C overnight. Tissue sections were then washed three times with 0.1 % Triton X-100 in PBS and incubated with Alexa Fluor 594-conjugated goat anti-rabbit secondary antibody (Life Technologies) for 1 h at room temperature. Slides were then rinsed in Triton/PBS and mounted with ProLong Diamond Antifade Mountant (Life Technologies). Images were acquired using a Zeiss Axio Imager M1 epifluorescence microscope using appropriate filters and a Plan-Apochromat 20× objective (NA 0.8) or a Plan-Apochromat 63× objective (NA 1.40) with oil immersion.

### Gel contraction assay

Compressible collagen matrices were prepared as previously indicated [62]. Gels were prepared containing type I collagen (Advanced BioMatrix) and 5 × DMEM, with pH adjusted to 7.0–7.4. Cardiac proto-myofibroblasts suspended in serum-supplemented DME/F-12 medium were seeded (5 × 10^5^ cells/well) on collagen gels for 24 h prior to equilibration by serum deprivation and adenovirus infection (AdGFP, AdScx, AdshLacZ or AdshScx at MOI 10). At initiation of contraction, gels were excised from wells using a custom-fabricated stainless steel dowel or a surgical blade (Thermo Fisher Scientific). Cells were treated with 10 ng/mL recombinant human TGF-β_1_ (R&D Systems) for 24 h. Well images were captured at 0 h and 24 h. Gel area for each well was determined using IDL Measure Gel software and reported as percentage of maximal (10 % serum-induced) contraction [63].

### Measurement of cardiomyocyte cross-sectional area

Cardiomyocyte cross-sectional area was measured as previously described [64, 65]. Six μm thick OCT-embedded cardiac tissue sections were treated with 3.3 U/mL neuraminidase type V (Sigma-Aldrich) for 1 h at room temperature. Sections were then rinsed with PBS and stained with fluorescein-labelled peanut agglutinin (Vector Laboratories) overnight at 4 °C. Tissue sections were rinsed with PBS three times and mounted with ProLong Diamond Antifade Mountant. The mountant was allowed to cure overnight in the dark. Images were acquired on a Zeiss Axio Imager M1 epifluorescence microscope with an EC Plan-Neofluar 40× objective (NA 0.75), and bounded area measured using AxioVision software. Fifty cells were analyzed from each animal.

### Determination of cardiac capillary density

Cardiac capillary density was assayed as previously described [66, 67]. Six μm thick left ventricle sections were treated with neuraminidase type V and stained with Rhodamine-labeled *Griffonia (Bandeiraea) simplicifolia* lectin I (GSL-I, BSL-I; Vector Laboratories) overnight at 4 °C. Slides were then washed with PBS, mounted using ProLong Diamond Antifade Mountant and allowed to cure overnight. Images were acquired as per measurements of cardiomyocyte cross-sectional area. Two fields were analyzed and averaged from each animal.

### Statistical analysis

Results are reported as mean ± standard error for at least three independent biological replicates for each sample; for studies involving animal tissue, each n represents one individual animal for each genotype. Means were compared by two-sided Student’s *t*-test, or for multiple comparisons by one-way ANOVA with Student-Newman-Keuls post-hoc analysis; results were considered significant when *P* < 0.05.

### Availability of data and materials

Data supporting the results of this article are available in Additional file 7.

## Additional files

Additional file 1: Figure S1. A scleraxis double deletion mutant fails to regulate target gene expression. (A) NIH-3T3 fibroblasts were transfected with a human COL1A2 proximal promoter luciferase reporter vector, with or without expression vectors for intact scleraxis (500 ng) or encoding a scleraxis mutant lacking the DNA- and protein-binding domains (ScxΔΔ; 0–500 ng), plus a Renilla luciferase reporter (pRL) for normalization. After 24 h, cell lysates were collected for luciferase assay. Intact scleraxis transactivated the COL1A2 promoter. In contrast, the ScxΔΔ mutant had no effect either alone or with co-transfected intact scleraxis. Results were normalized to control (C; reporter alone); mean ± SEM; *n* = 3; **P* < 0.05 vs control. (B, C) Primary cardiac proto-myofibroblasts were transfected by electroporation with an expression vector encoding the scleraxis double mutant (pScxΔΔ) or with control empty vector (pECE). Then, 24 h later, mRNA expression of fibrillar collagen gene expression (B) or (myo)fibroblast markers (C) was assayed by qPCR. Results were normalized to the control empty vector and *Gapdh* expression; mean ± SEM; *n* = 3; *P* > 0.05 for all comparisons (B–C). (EPS 1153 kb) Additional file 2: Table S1. Echocardiographic parameters. **Table S2.** Antibodies used in the study. **Table S3.** Primers used for qPCR analysis. **Table S4.** Primers used for site-directed mutagenesis of αSMA proximal promoter. **Table S5.** Primers used for nested PCR generation of ScxΔΔ. **Table S6.** Probes used for electrophoretic mobility shift assay. **Table S7.** Primers used for chromatin immunoprecipitation assay. (DOCX 34 kb) Additional file 3: Figure S2. Flow cytometry analysis of cardiac cells from WT and scleraxis KO mice. (A) Scatter profile shows cellular aggregates and tissue debris being gated out using side-scatter area (SSC-A) and forward-scatter area (FSC-A). (B) Forward-scatter plots for 5 × 10^4^ events labeled for αMHC, αSMA, DDR2 or CD31; left column, unstained cells; center column, stained cells from WT tissue; right column, stained cells from scleraxis KO tissue. Results are representative of assessments from *n* = 3 independent tissue samples. Purple outline denotes labeled cells, and is derived from unstained plots. (EPS 7579 kb) Additional file 4: Figure S3. Capillary density and cardiomyocyte cross-sectional area analysis of cardiac sections from WT and scleraxis KO mice. (A) Cardiac tissue sections (6 μm) from WT and scleraxis KO mice were stained with Rhodamine-labeled GSL I and capillaries counted to assess density. (B) Results for *n* = 3 independent samples per genotype (two fields/sample); mean ± SEM; **P* < 0.05 vs WT. (C) Cardiac tissue sections (6 μm) from WT and scleraxis KO mice were stained with fluorescein-labeled peanut agglutinin for measurement of cardiomyocyte cross-sectional area. (D) Results for *n* = 3 independent samples per genotype (50 cells/sample); mean ± SEM; **P* < 0.05 vs WT; 40× objective, scale bar = 25 μm. (EPS 19611 kb) Additional file 5: Figure S4. LIVE/DEAD assay of primary cardiac fibroblasts following scleraxis knockdown. (A) Primary adult rat cardiac fibroblasts were assessed for viability by LIVE/DEAD assay following treatment with AdshLacZ or AdshScx for 48 h. Viable cells are green, while dead cells are red (arrowheads); 200 μM H_2_O_2_ was used as a positive control for cell death. (B) Results from (A) were quantified and reported as percent of dead cells out of total cells (789–1,660 cells counted per biological replicate; 4–6 fields of cells were collectively assessed for AdshLacZ and AdshScx samples; all cells died following H_2_O_2_ treatment; 417–541 cells counted per H_2_O_2_ replicate); mean ± SEM; *n* = 3; *P* > 0.05 for AdshLacZ vs AdshScx; 10× objective, scale bar = 100 μm. (EPS 16834 kb) Additional file 6: Figure S5. Induction of fibroblast, mesenchyme and tendon markers in C3H10T1/2 cells by scleraxis. C3H10T1/2 cells were treated with AdGFP or AdScx for 48 h, and mRNA assayed by qPCR for representative fibroblast, mesenchyme and tendon marker gene expression. Results were normalized to *Gapdh* and AdGFP-infected samples; mean ± SEM; *n* = 3; **P* < 0.05 vs AdGFP. (EPS 978 kb) Additional file 7: Supporting data. (XLSX 284 kb)

## Abbreviations

αSMA: α-smooth muscle actin
DDR2: discoidin domain receptor 2
ECM: extracellular matrix
EDA-Fn: extra domain-A splice variant of fibronectin
EMT: epithelial-to-mesenchymal transition
FB: fibroblast
KO: knockout
MFB: myofibroblast
MMP: matrix metalloproteinase
pMFB: proto-myofibroblast
Scx: scleraxis
SM_emb_: embryonic smooth muscle myosin
WT: wild type
