# Supplementary material for: The transcription factor scleraxis is a critical regulator of cardiac fibroblast phenotype
Source: BMC Biol. 2016 Mar 17;14:21. doi: 10.1186/s12915-016-0243-8 (PMC4794909; doi:10.1186/s12915-016-0243-8)
Supplement: Additional file 2: Table S1. — Echocardiographic parameters. Table S2. Antibodies used in the study. Table S3. Primers used for qPCR analysis. Table S4. Primers used for site-directed mutagenesis of αSMA proximal promoter. Table S5. Primers used for nested PCR generation of ScxΔΔ. Table S6. Probes used for electrophoretic mobility shift assay. Table S7. Primers used for chromatin immunoprecipitation assay. (DOCX 34 kb) [file 12915_2016_243_MOESM2_ESM.docx]

**SUPPLEMENTAL DATA**

**Table S1. Echocardiographic parameters.**

| **Parameter** | **Wild type** | **Scleraxis null** |
| --- | --- | --- |
| LVEF (%) | 80±1 | 80±1 |
| HR (bpm) | 671±9 | 679±10 |
| FS (%) | 50±1 | 50±1 |
| LVEDD/TL | 0.153±0.003 | 0.166±0.004* |
| LVESD/TL | 0.077±0.002 | 0.082±0.003 |
| PWT/TL | 0.048±0.001 | 0.047±0.001 |
| IVS/TL | 0.047±0.001 | 0.045±0.001 |
| Vendo (cm/s) | 2.2±0.08 | 2.0±0.06 (P=0.0541) |
| SR (s^-1^) | 18±1 | 17±1 |
| MV E (m/s) | 929±79 | 882±85 |
| MV A (m/s) | 544±35 | 618±79 |
| E/A | 1.71±0.22 | 1.81±0.27 |
| DT (msec) | 27±2 | 28±5 |

*P<0.05

LVEF, left ventricular ejection fraction; HR, heart rate; FS, fractional shortening; TL, tibia length; LVEDD, left ventricular end diastolic diameter; LVESD, left ventricular end systolic diameter; PWT, posterior wall thickness; IVS, interventricular septal thickness; Vendo, endocardial velocity; SR, strain rate ; MV E, early diastolic flow velocity; MV A, late diastolic inflow velocity; E/A, ratio of the mitral E to A waves; DT, deceleration time.

**Table S2. Antibodies used in the study.**

Abbreviations: ChIP, chromatin immunoprecipitation; DSHB, Developmental Studies Hybridoma Bank; FACS, flow-activated cell sorting; IF, immunofluorescence; IP, immunoprecipitation; WB, western blotting

| **Antibody** | **Application** | **Dilution** | **Vendor** | **RRID/Catalog#** |
| --- | --- | --- | --- | --- |
| Type I collagen | WB | 1:1000 | Cedarlane | AB_10060024 |
|  | IF | 1:100 |  |  |
| Type III collagen | WB | 1:1000 | DSHB Iowa | AB_528166 |
| Type IV collagen | WB | 1:1000 | DSHB Iowa | AB_528167 |
| α-tubulin (12G10) | WB | 1:5000 | DSHB Iowa | AB_1157911 |
| GAPDH | WB | 1:5000 | Sigma | G8795 |
| DDR2 | WB | 1:1000 | Sigma | SAB1302555 |
|  | IF | 1:100 |  |  |
|  | FACS | 1:50 |  |  |
| αSMA | WB | 1:5000 | Sigma | AB_476701 |
|  | IF | 1:500 | Abcam | AB_306359 |
|  | FACS | 1:50 |  |  |
| Vimentin | WB | 1:1000 | Abcam | AB_883448 |
| SMemb | WB | 1:1000 | Abcam | AB_305661 |
| ED-A fibronectin | WB | 1:1000 | Millipore | AB_94436 |
| β-actin | WB | 1:1000 | Cell Signaling | AB_10695744 |
| Twist1 | IF | 1:100 | Sigma | AB_609890 |
| αMHC | FACS | 1:50 | Abcam | AB_2250954 |
| CD31 (PE/Cy7) | FACS | 1:50 | BD Biosciences | AB_10612003 |
| Scleraxis | WB | 1:1000 | Custom polyclonal  (QED Bioscience) | * |
|  | ChIP | 10 µg |  |  |
| HA | WB | 1:1000 | Rockland Immunochemicals | AB_217929 |
|  | IP | 5 µg |  |  |
| c-Myc (9E10) | WB | 1:1000 | DSHB Iowa | AB_2266850 |
| Smad3 | ChIP | 5 µg | Abcam | AB_2192903 |
| RNA Polymerase II | ChIP | 1 µg | Millipore | AB_309852 |

*Described in Espira L, Lamoureux L, Jones SC, Gerard RD, Dixon IM, Czubryt MP: **The basic helix-loop-helix transcription factor scleraxis regulates fibroblast collagen synthesis.** *J Mol Cell Cardiol* 2009, **47**(2):188-195.

**Table S3. Primers used for qPCR analysis.**

| **Amplicon** | **Direction** | **Sequence (5’🡪 3’)** |
| --- | --- | --- |
| Biglycan (Rat) | Forward | CTTCCGCTGCGTCACTGA |
|  | Reverse | GGTGGCTACCACTGCTTCTACTTC |
| Biglycan (Mouse) | Forward | ATTGCCCTACCCAGAACTTGAC |
|  | Reverse | GCAGAGTATGAACCCTTTCCTG |
| Claudin1 (Mouse) | Forward | TGGGGCTGATCGCAATCTTT |
|  | Reverse | ATGGGGGTCAAGGGGTCATA |
| Claudin1 (Rat) | Forward | TGGGGCTGATCGCAATCTTT |
|  | Reverse | TGATAGGGGTCATGGGGTCA |
| Claudin1 (Human) | Forward | CCTTCCTCTCTCTACCAGTCTAT |
|  | Reverse | GGAATTACAAACCACCGCTTAC |
| Col5α1 (Rat) | Forward | CACTCGACGATCTTCCAAAG |
|  | Reverse | TCAGGATGGAGAAGTCCTC |
| Col5α1 (Mouse) | Forward | GGACTAGTCCGCTTTCCCTGTCAACTTGTCCGATGG |
|  | Reverse | GTGGTCACTGCGGCTGAGGAACTTC |
| Decorin (Rat/Mouse) | Forward | CCTGACAATCCCCTGATATCTATGT |
|  | Reverse | GTCCAGACCCAGATCAGAACACT |
| Desmoplakin (Mouse) | Forward | GCAGAAGGAAGACGATTCCAAGA |
|  | Reverse | TTCCGAGCCACAGGCTTTC |
| Desmoplakin (Rat) | Forward | AGCCCTTCTCCAGGCAATTC |
|  | Reverse | GGAGTGAATCTGCTGTGCCT |
| Desmoplakin (Human) | Forward | TACTGGTCTGCTCTTGCTTTC |
|  | Reverse | CTCTCACCAACCTCGTCATAAG |
| Fibromodulin (Rat) | Forward | GCTCTGGGCTCCTACTCCTT |
|  | Reverse | GTCCTGCCATTCTGAGGTGT |
| DDR2 (Rat/Mouse) | Forward | GATCATGTTTGAATTTGACCGA |
|  | Reverse | GCACTGGGGTTCACATC |
| ED-A-Fibronectin (Rat/Mouse) | Forward | ACTGCAGTGACCAACATTGACC |
|  | Reverse | CACCCTGTACCTGGAAACTTGC |
| ED-A-Fibronectin (Human) | Forward | CAGGCCACTTAAACTCCTACA |
|  | Reverse | CCTCCTCACTCAGCTCATATTC |
| Fibromodulin (Mouse) | Forward | CAATGTCTACACCGTCCCTGA |
|  | Reverse | AGAAGGCTGCTGGAGTTGAAG |
| Lumican (Rat) | Forward | GTTGAAAAGTGTGCCCATGGT |
|  | Reverse | TTCATCAATATGGTCGATCTGGTT |
| Lumican (Mouse) | Forward | AGATGCTTGATCTTGGAGTAAGA |
|  | Reverse | CAATGAACTTGAAAAGTTTGATG |
| MMP2 (Rat/Mouse) | Forward | CCCATGAAGCCTTGTTTACCA |
|  | Reverse | TGGAAGCGGAACGGAAACT |
| MMP3 (Rat) | Forward | AGGTCATGAAGAGCTAGCAG |
|  | Reverse | CAGGAGTGTGTTTTCTCCTC |
| MMP3 (Mouse) | Forward | CTATACGAGGGCACGAGGAG |
|  | Reverse | CCACCCTTGAGTCAACACCT |
| MMP9 (Rat) | Forward | CGTGGCCTACGTGACCTATGA |
|  | Reverse | TGCACCGCTGAAGCAAAAG |
| MMP9 (Mouse) | Forward | TGTCTGGAGATTCGACTTGAAGTC |
|  | Reverse | TGAGTTCCAGGGCACACCA |
| MMP11 (Rat) | Forward | AAGTTTCCCTCGACCCATAGG |
|  | Reverse | AGGTGTTGTCAGCGGAAAGTG |
| MMP11 (Mouse) | Forward | CCTTCCAGGATGCTGAGGGCTAT |
|  | Reverse | ATGACAGCATGGTCGTTCCTACAA |
| Par3 (Mouse) | Forward | TCCTGCTGTTTGTGGTTGGT |
|  | Reverse | GTGACCTCGCCAAATACCCA |
| Par3 (Rat) | Forward | AGCGAAGTGTCCCGAAGAAA |
|  | Reverse | ATGGCCAGGTTGGTGTGAAA |
| Par3 (Human) | Forward | CTTCACAAGTCCTGCCAGATTA |
|  | Reverse | TCTGAGGCAGAGTGGAGATATAG |
| Periostin (Rat) | Forward | TCGTGGAACCAAAAATTAAAGTC |
|  | Reverse | CTTCGTCATTGCAGGTCCTT |
| Periostin (Mouse) | Forward | TGCTGCCCTGGCTATATGAG |
|  | Reverse | GTAGTGGCTCCCACAATGCC |
| αSMA (Human) | Forward | GATCTGGCACCACTCTTTCTAC |
|  | Reverse | ATTGTGGGTGACACCATCTC |
| SMemb (Rat/Mouse) | Forward | CAATGAGGGTGGGAGTGGGTG |
|  | Reverse | CCTCTCGGGATCCTCCAGTC |
| Snai1 (Mouse) | Forward | AGTTGACTACCGACCTTGCG |
|  | Reverse | TGCAGCTCGCTATAGTTGGG |
| Snai1 (Rat) | Forward | GGAAGCTTGAACCCCACTCA |
|  | Reverse | CCACTTGGCCCCTAACAAGT |
| Snai1 (Human) | Forward | GACCCACTCAGATGTCAAGAAG |
|  | Reverse | CATGGCAGTGAGAAGGATGT |
| TCF21 (Mouse) | Forward | CATTCACCCAGTCAACCTGA |
|  | Reverse | CCACTTCCTTCAGGTCATTCTC |
| Tenomodulin (Mouse) | Forward | TGTACTGGATCAATCCCACTCT |
|  | Reverse | GCTCATTCTGGTCAATCCCCT |
| Tenomodulin (Human) | Forward | ACTTTGAGGAGGAGGGAGAA |
|  | Reverse | CCTCGACGGCAGTAAATACAA |
| Twist1 (Mouse/Rat) | Forward | CAGCGGGTCATGGCTAACG |
|  | Reverse | ATCTTGCTCAGCTTGTCCGA |
| Twist1 (Human) | Forward | CATCCTCACACCTCTGCATT |
|  | Reverse | AGTCCATAGTGATGCCTTTCC |
| Versican (Rat/Mouse) | Forward | CTGATAGCAGATTTGATGCCTACTGC |
|  | Reverse | GTGGTTCTTTGGATAAACTGGGTGATG |
| Vimentin (Rat/Mouse) | Forward | ACATCCACCCGCACCTAC |
|  | Reverse | CAACTCCCTCATCTCCTCCTC |
| Vimentin (Human) | Forward | GCTCGTCACCTTCGTGAATA |
|  | Reverse | TCGTTGATAACCTGTCCATCTC |
| Zeb1 (Mouse) | Forward | GCGGCGCAATAACGTTACAA |
|  | Reverse | GGGCGCCTCAGGATAAATGA |
| Zeb1 (Rat) | Forward | GCGGCGCAATAACGTTACAA |
|  | Reverse | TCCACGTTGGGATCATGGTT |
| Zeb1 (Human) | Forward | GACCTCATGAGTGTGGAATCTG |
|  | Reverse | CGTTCTTCCGCTTCTCTCTTAC |

**Table S4. Primers used for site-directed mutagenesis of αSMA proximal promoter.**

Mutated E boxes are underlined.

| **Amplicon** | **Direction** | **Sequence (5’🡪 3’)** |
| --- | --- | --- |
| E box 1 | Forward | GTCTGGGCATTTGAGCCGATGTTCTGAGGGCTCAGG |
|  | Reverse | CCTGAGCCCTCAGAACATCGGCTCAAATGCCCAGAC |
| E box 2 | Forward | GTTTATCCCCATAAGCTGGTGAACTGCCTCCTGTTT |
|  | Reverse | AAACAGGAGGCAGTTCACCAGCTTATGGGGATAAAC |

**Table S5. Primers used for nested PCR generation of ScxΔΔ.**

| **Amplicon** | **Direction** | **Sequence (5’🡪 3’)** |
| --- | --- | --- |
| ScxΔΔ | Forward | GGGCTCGCGGCCTGGC |
|  | Reverse | AATGTGCTGCTGGTGGGTGAGGC |

**Table S6. Probes used for electrophoretic mobility shift assay.**

E boxes are underlined.

| **Amplicon** | **Direction** | **Sequence (5’🡪 3’)** |
| --- | --- | --- |
| E box 1 (E1) | Forward | GTCTGGGCATTTGAGCAGTTGTTCTGAGGGCTCAGG |
|  | Reverse | CCTGAGCCCTCAGAACAACTGCTCAAATGCCCAGAC |
| E box 2 (E2) | Forward | GTTTATCCCCATAAGCAGCTGAACTGCCTCCTGTTT |
|  | Reverse | AAACAGGAGGCAGTTCAGCTGCTTATGGGGATAAAC |
| E box 1 mutant (mE1) | Forward | GTCTGGGCATTTGAGCCGATGTTCTGAGGGCTCAGG |
|  | Reverse | CCTGAGCCCTCAGAACATCGGCTCAAATGCCCAGAC |
| E box 2 mutant (mE2) | Forward | GTTTATCCCCATAAGCTGGTGAACTGCCTCCTGTTT |
|  | Reverse | AAACAGGAGGCAGTTCACCAGCTTATGGGGATAAAC |

**Table S7. Primers used for chromatin immunoprecipitation assay.**

For the αSMA gene promoter, E boxes 1 and 2 are 32 nucleotides apart, thus a single set of primers was used.

| **Amplicon** | **Direction** | **Sequence (5’🡪 3’)** |
| --- | --- | --- |
| αSMA E box 1/2 | Forward | aaaggctagcctgacaggaagagc |
|  | Reverse | gggacctcagcacaaaactctctaatc |
| Col1α2-SBE (Mouse) | Forward | GGAGATCTGTAAAGAGCCCACGTA |
|  | Reverse | AGGGACGTGGCTACGGGGCTTCTTA |
| COL1A2-SBE (Human) | Forward | ATTCTGCCCATGTCGGGGCTGCA |
|  | Reverse | GAGGAGGGAGCGAATGGGGGAA |
| Gapdh (Rat/Mouse) | Forward | aaacaagttcaccaccatgtgaaa |
|  | Reverse | ccagggattgaccaaaggtgagtt |
